# Cardiovascular/Stroke Risk Stratification in Diabetic Foot Infection Patients Using Deep Learning-Based Artificial Intelligence: An Investigative Study

**DOI:** 10.3390/jcm11226844

**Published:** 2022-11-19

**Authors:** Narendra N. Khanna, Mahesh A. Maindarkar, Vijay Viswanathan, Anudeep Puvvula, Sudip Paul, Mrinalini Bhagawati, Puneet Ahluwalia, Zoltan Ruzsa, Aditya Sharma, Raghu Kolluri, Padukone R. Krishnan, Inder M. Singh, John R. Laird, Mostafa Fatemi, Azra Alizad, Surinder K. Dhanjil, Luca Saba, Antonella Balestrieri, Gavino Faa, Kosmas I. Paraskevas, Durga Prasanna Misra, Vikas Agarwal, Aman Sharma, Jagjit S. Teji, Mustafa Al-Maini, Andrew Nicolaides, Vijay Rathore, Subbaram Naidu, Kiera Liblik, Amer M. Johri, Monika Turk, David W. Sobel, Martin Miner, Klaudija Viskovic, George Tsoulfas, Athanasios D. Protogerou, Sophie Mavrogeni, George D. Kitas, Mostafa M. Fouda, Mannudeep K. Kalra, Jasjit S. Suri

**Affiliations:** 1 Department of Cardiology, Indraprastha APOLLO Hospitals, New Delhi 110001, India; 2 Stroke Monitoring and Diagnostic Division, AtheroPoint™, Roseville, CA 95661, USA; 3 Department of Biomedical Engineering, North Eastern Hill University, Shillong 793022, India; 4 MV Diabetes Centre, Royapuram, Chennai 600013, India; 5 Annu’s Hospitals for Skin and Diabetes, Nellore 524101, India; 6 Max Institute of Cancer Care, Max Super Specialty Hospital, New Delhi 110017, India; 7 Invasive Cardiology Division, Faculty of Medicine, University of Szeged, 6720 Szeged, Hungary; 8 Division of Cardiovascular Medicine, University of Virginia, Charlottesville, VA 22904, USA; 9 Ohio Health Heart and Vascular, Columbus, OH 43214, USA; 10 Neurology Department, Fortis Hospital, Bangalore 560076, India; 11 Heart and Vascular Institute, Adventist Health St. Helena, St Helena, CA 94574, USA; 12 Department of Physiology & Biomedical Engineering, Mayo Clinic College of Medicine and Science, Rochester, MN 55905, USA; 13 Department of Radiology, Mayo Clinic College of Medicine and Science, Rochester, MN 55905, USA; 14 Department of Radiology, Azienda Ospedaliero Universitaria, 40138 Cagliari, Italy; 15 Cardiovascular Prevention and Research Unit, Department of Pathophysiology, National & Kapodistrian University of Athens, 15772 Athens, Greece; 16 Department of Pathology, Azienda Ospedaliero Universitaria, 09124 Cagliari, Italy; 17 Department of Vascular Surgery, Central Clinic of Athens, 15772 Athens, Greece; 18 Department of Immunology, SGPGIMS, Lucknow 226014, India; 19 Ann and Robert H. Lurie Children’s Hospital of Chicago, Chicago, IL 60611, USA; 20 Allergy, Clinical Immunology and Rheumatology Institute, Toronto, ON L4Z 4C4, Canada; 21 Vascular Screening and Diagnostic Centre, University of Nicosia Medical School, Egkomi 2408, Cyprus; 22 AtheroPoint™, Roseville, CA 95661, USA; 23 Electrical Engineering Department, University of Minnesota, Duluth, MN 55812, USA; 24 Department of Medicine, Division of Cardiology, Queen’s University, Kingston, ON K7L 3N6, Canada; 25 The Hanse-Wissenschaftskolleg Institute for Advanced Study, 27753 Delmenhorst, Germany; 26 Rheumatology Unit, National Kapodistrian University of Athens, 15772 Athens, Greece; 27 Men’s Health Centre, Miriam Hospital Providence, Providence, RI 02906, USA; 28 Department of Radiology and Ultrasound, University Hospital for Infectious Diseases, 10000 Zagreb, Croatia; 29 Department of Surgery, Aristoteleion University of Thessaloniki, 54124 Thessaloniki, Greece; 30 Cardiology Clinic, Onassis Cardiac Surgery Centre, 17674 Athens, Greece; 31 Academic Affairs, Dudley Group NHS Foundation Trust, Dudley DY1 2HQ, UK; 32 Arthritis Research UK Epidemiology Unit, Manchester University, Manchester M13 9PL, UK; 33 Department of Electrical and Computer Engineering, Idaho State University, Pocatello, ID 83209, USA; 34 Department of Radiology, Harvard Medical School, Boston, MA 02115, USA

**Keywords:** diabetics, diabetic’s foot infection, cardiovascular/stroke risk stratification, deep learning, AI bias

## Abstract

A diabetic foot infection (DFI) is among the most serious, incurable, and costly to treat conditions. The presence of a DFI renders machine learning (ML) systems extremely nonlinear, posing difficulties in CVD/stroke risk stratification. In addition, there is a limited number of well-explained ML paradigms due to comorbidity, sample size limits, and weak scientific and clinical validation methodologies. Deep neural networks (DNN) are potent machines for learning that generalize nonlinear situations. The objective of this article is to propose a novel investigation of deep learning (DL) solutions for predicting CVD/stroke risk in DFI patients. The Preferred Reporting Items for Systematic reviews and Meta-Analyses (PRISMA) search strategy was used for the selection of 207 studies. We hypothesize that a DFI is responsible for increased morbidity and mortality due to the worsening of atherosclerotic disease and affecting coronary artery disease (CAD). Since surrogate biomarkers for CAD, such as carotid artery disease, can be used for monitoring CVD, we can thus use a DL-based model, namely, Long Short-Term Memory (LSTM) and Recurrent Neural Networks (RNN) for CVD/stroke risk prediction in DFI patients, which combines covariates such as office and laboratory-based biomarkers, carotid ultrasound image phenotype (CUSIP) lesions, along with the DFI severity. We confirmed the viability of CVD/stroke risk stratification in the DFI patients. Strong designs were found in the research of the DL architectures for CVD/stroke risk stratification. Finally, we analyzed the AI bias and proposed strategies for the early diagnosis of CVD/stroke in DFI patients. Since DFI patients have an aggressive atherosclerotic disease, leading to prominent CVD/stroke risk, we, therefore, conclude that the DL paradigm is very effective for predicting the risk of CVD/stroke in DFI patients.

## 1. Introduction

Foot ulcers are the leading cause of morbidity and amputation in people with diabetes. These complications also contribute to significant healthcare expenditure, as indicated by the fact that 20 to 40% of healthcare resources are spent on diabetic feet associated with diabetes [1,2]. As per the World Health Organization (WHO), diabetic foot syndrome (DFS) is described as “ulceration of the foot (distally from the ankle and including the ankle) linked with neuropathy and various grades of ischemia and infection” [3]. It is a severe long-term complication of diabetes mellitus (DM) that can lead to disability, amputations, cardiovascular diseases, and a lower quality of life [4,5].

In the United States, approximately 73,000 lower-extremity amputations are carried out each year due to diabetes [6]. Foot ulceration is the primary and sole factor that causes 80% of these complications [7,8]. The existence of foot ulceration is believed to be a significant risk factor for morbidity, death, and disability. This notion is confirmed by the fact that the diabetic condition is responsible for approximately 80% of nontraumatic amputations and that 85% of these amputations are preceded by foot ulceration [9]. It is thought that 15% of diabetics will get an ulcer on one of their lower limbs at some point during their disease [10]. A connection between a diabetic foot infection (DFI) and cardiovascular disease (CVD) has been discovered by several investigations [11,12,13]. DFI is an indicator of diabetes, and when active and uncontrolled, raises the risk of CVD [14,15,16].

The greatest risk factors for coronary heart disease (CHD) and diabetes include obesity, high blood pressure, and high blood cholesterol [17,18]. The diabetic foot ulcer (DFU) disease also causes inflammatory reactions, which can contribute to the development of atherosclerosis, promoting coronary artery disease (CAD), and the worsening of CVD [19,20,21,22,23]. Multiple studies relate more advanced stages of a DFI to more severe forms of atherosclerotic cardiovascular disease (ASCVD) [15,23,24,25]. As a result, a DFI contributes to the development of CVD. It is essential to understand the connection between a DFI and CVD to reduce the risk of heart attacks, cardiovascular events (CVE), and stroke [9,26].

The development of calcifications and hemorrhagic formation characteristics, as seen in a DFI, increases the risk of CVD [27,28]. Foot wound imaging is an essential procedure in examining a DFI [29]. It is essential to use foot imaging to monitor changes in a DFI to provide an accurate assessment of the prevalence of diabetics [30]. It is suggested that coronary imaging be performed to determine the risk of developing CVD [23]. In addition, imaging of the coronary arteries is necessary to identify plaque in CAD [31,32]. Intravenous ultrasonography (IVUS) and optical coherence tomography (OCT) are two examples of effective imaging technologies that can be used to diagnose coronary plaque [33,34,35]. Since surrogate markers are well established for CAD, such as carotid artery imaging and its quantification, thus, there is a need for (i) accurate and computerized carotid plaque load assessment, (ii) effective detection of atherosclerotic disease in DFI patients and (iii) CVD risk stratification. All three aspects are essential to prevent DFI-driven CVD from becoming severe. Hence, there is a need for the automated and early assessment of a diabetic foot infection (DFI) and CVD severity in patients to avoid morbidity and mortality.

Artificial intelligence (AI) has fundamentally altered the dynamics of the healthcare sector [36]. Machine Learning (ML) and Deep Learning (DL) algorithms have been implemented in a variety of medical applications [37,38]. AI-based technologies are data-driven, which means they make decisions based on information in databases, and have been used to diagnose diabetes [39,40], liver [41], thyroid [42], and skin cancer [43], just to name a few. Regarding CVD, the results show that there are nonlinear connections between the input predictors and the cardiovascular outcomes [44,45]. In contrast to the statistical risk estimation techniques currently in use [44,46], ML-based algorithms may use intricate quasi-relationships among several risk predictors (or attributes) that are input simultaneously.

DL algorithms extract characteristics directly from the input data to generate predictions. Some examples include the characterization of carotid wall tissue, the segmentation of pictures, and the stratification of CVD risk [47,48]. It has also been established that DL algorithms with convolution neural networks (CNNs) extract features, which can then be used to train and test an ML classifier to obtain a final classification [49,50]. Recently, images of the DFI foot wound have been utilized to predict the severity of the disease. It has been demonstrated that algorithms based on ML and DL can accurately predict a DFI [29,30]. Because of this, it is conceivable for AI-based solutions to allow the analysis of image-based diabetic foot inputs [51]. This is made possible by eliminating the demand for human intervention. Several applications of carotid ultrasonography that use AI-based algorithms have shown a lot of promise [52,53,54]. Thus, it means that these AI-based methods could be used to evaluate a patient’s risk and treat both DFI and CVD disorders concurrently.

The usage of alternative imaging for the visualization of CAD helps in the categorization of DFI patients into appropriate CVD risk categories [55,56,57]. This is because CAD is easier to see with surrogate imaging. Thus, to gain a more in-depth insight into the pathophysiology of diabetes, diabetes foot ulcer, and cardiovascular disease, this study focuses on the use of low-cost carotid artery and diabetic foot ultrasound imaging. Using techniques such as ML and DL, it is possible to identify patients who are at significant risk of developing CVD complications [58]. To best analyze the above study, we have adopted the search strategy and the distributions.

## 2. Search Strategy Using PRISMA Model

**Figure 1:**
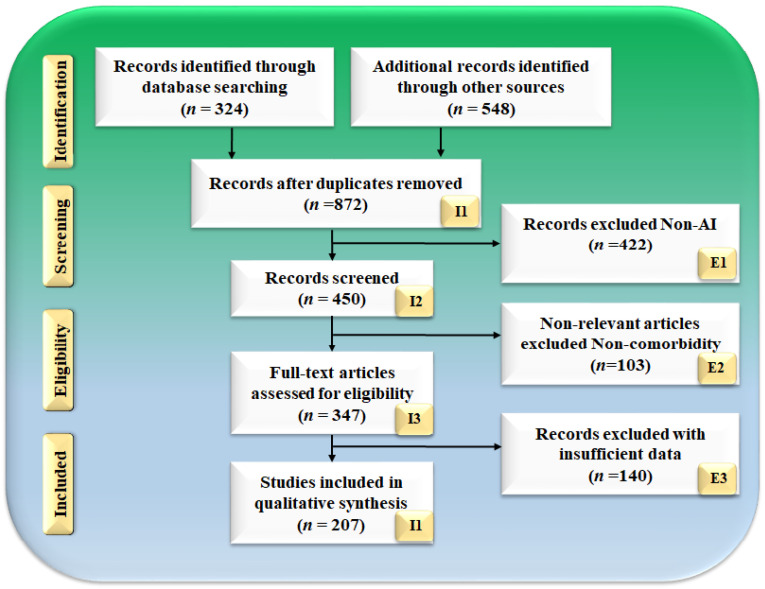
PRISMA model for selection of studies.

**Figure 2:**
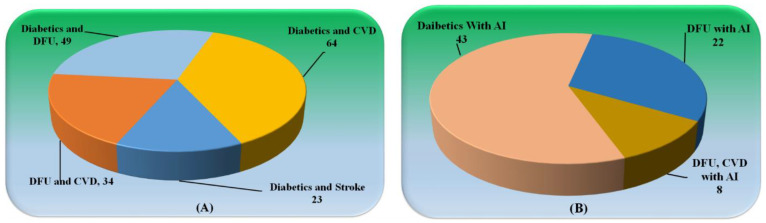
(**A**) Studies related to Diabetics with CVD, Stroke, and DFU. (**B**) Studies explaining the role of AI in Diabetics with DFU and CVD.

The Preferred Reporting Items for Systematic reviews and Meta-Analyses (PRISMA) model (Figure 1) is used as the basis for the search method. PubMed, IEEE, and Google Scholar are three databases that are used to search for and screen relevant papers. These databases are searched with keywords such as “diabetic foot ulcer”, “diabetic foot disease”, “diabetic foot infection”, “diabetes”, “CVD”, “diabetic foot ulcer and CVD”, “diabetic foot ulcer and coronary artery disease”, “diabetic foot imaging”, “diabetes and carotid imaging”, “artificial intelligence”, “artificial intelligence and CVD”, “machine learning and CVD”, “deep learning and CVD”, “classifiers and CVD/stroke risk stratification”, and “atherosclerotic plaque tissue classification”. There was a total of 324 papers located on PubMed, and there were 548 articles initially selected from Google Scholar and IEEE. To narrow the list down to just 872 articles, sophisticated criteria such as time and relevancy were utilized. After considering whether or not to include them in this evaluation, a total of 140 articles were narrowed down to the articles that made the final list. The following are the three criteria that were used to exclude studies: (i) studies that did not relate in any way to our study objective, (ii) papers that did not contain useful information, and (iii) studies that contained insufficient data in the studies. Following the elimination of 422, 103, and 140 investigations (respectively denoted with the letters E1, E2, and E3), a final pool of 207 studies was chosen for the final analysis out of a total of 450 studies. Figure 2 depicts the comprehensive screening procedure for the selection of the research paper.

### Statistical Distribution

Figure 2a shows the studies related to (i) diabetes and DFU, (ii) diabetes and CVD, (iii) DFU and CVD, and diabetes and stroke. A number of the articles explain the role of diabetics leading to the development of CVD in a patient. Figure 2b shows the distribution of studies of AI with (i) Diabetics, (ii) DFU, and (iii) DFU and CVD. Each study had an examination utilizing a feasibility analysis, which was followed by a cross-check using scientific validation to guarantee that it came as close as possible to meeting our goals.

## 3. Pathobiological Mechanisms of Diabetes, CVD, and Diabetic Foot

**Figure 3:**
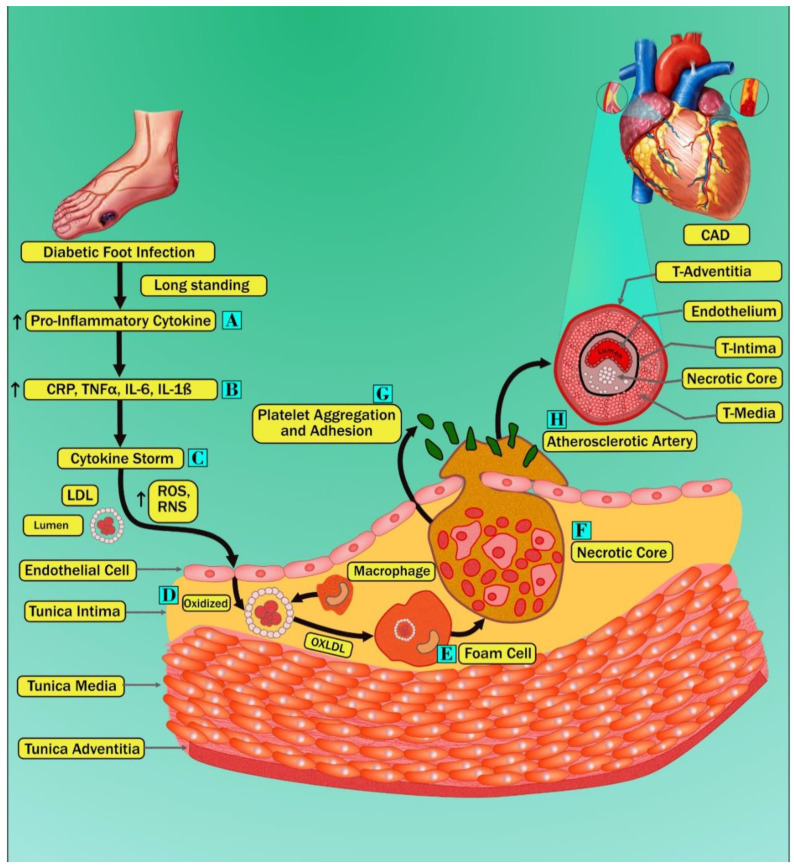
Pathobiological mechanisms of diabetes mellitus, cardiovascular disease, and diabetic foot are shown by different stages marked as A–H.

Figure 3 shows the biological link between diabetes mellitus and CVD. The survival rate of diabetic patients is lower than that of nondiabetic patients [59]. In the context of CVD, many studies showed that diabetes patients had 2–4 folds increased morbidity and mortality rates than patients without diabetes mellitus (DM) [60]. In addition, DM patients suffering from a foot infection have increased morbidity and mortality rates due to CVD about twice as much compared to patients with DM without a foot disease. A paper published by Pinto et al. [61] demonstrated an increased risk of CVD morbidity and mortality in DM patients who experienced amputation due to a foot infection compared to DM patients without a foot disease. Furthermore, in this study, authors also mentioned that patients suffering from a DFI have higher levels of serum cholesterol, serum triglycerides, and microalbuminuria or proteinuria, which are considered CV risk factors, compared with DM patients without a foot infection [62,63,64]. Another recent five-year follow-up study showed an increased risk of cerebrovascular events in DM patients with a foot disease compared to DM patients without a foot disease [25]. The published works [62,63,64] demonstrate that patients with a DFI are more prone to increased mortality and morbidity due to CVD than diabetic patients without a foot disease. We, thus, hypothesize that longstanding nonhealing ulcers in diabetes patients result in the activation of cytokine production, which further damages the heart (stage A of Figure 3). Interestingly, supporting our hypothesis, Jeffocate et al. [65], in their recent article, specified that patients with a DFI are more prone to developing an inflammatory cascade of increased levels of proinflammatory cytokines such as interleukin-1beta (IL-1β) and tumor necrosis factor-alpha (TNF- α), compared with diabetic patients without foot diseases. Additionally, Weigelt et al. [66] also showed that a DFI is responsible for the increase in circulation of acute phase cytokines such as interleukin 6 (IL6) and C-reactive protein (CRP). The above evidence demonstrated that immune activation in chronic nonhealing wounds is the key source of developing CV risk factors in patients with DM (stage A of Figure 3). These increased proinflammatory cytokines due to immune activation can trigger intracellular and extracellular reactive oxygen species (ROS). Furthermore, (stage C of Figure 3) results in damage to endothelial cells and causes the opening of inter endothelial junctions in a blood vessel [67]. Thus, this damage in the endothelium layer results in the penetration of native low-density lipoprotein (LDL) particles inside the tunica intimal layer, and this process is known as transcytosis [68]. Oxidative stress due to increased levels of ROS results in the formation of oxidized LDL (OxLDL), formed by the peroxidation of phospholipid molecules on the surface of LDL particles (Stage D of Figure 3). This process is known as lipid peroxidation [69]. Due to the presence of cellular and humoral innate immunity, OxLDL is taken by the macrophage, and this triggers the accumulation of many OxLDL inside the macrophage, resulting in the development of foam cells (stage E of Figure 3) [70,71]. Excess accumulation of foam cells increases the intake of more cholesterol, causing apoptosis and necrosis and progressing to the formation of the necrotic core (stage F of Figure 3) [72,73]. These attract the aggregation and adhesion of platelets, resulting in the development of atherosclerotic plaque (stage G and H of Figure 3) [74].

The endogenous and exogenous metabolic disruptions concerning glucose metabolism and their respective molecular repercussions contribute to an elevated risk of cardiovascular disease in patients with diabetes. The revelation of the cardiovascular outcome trial (CVOT) data and the discovery of certain unexpected advantages of major adverse cardiovascular events (MACE) in these trials highlight that higher levels might have both direct and indirect impacts. The metabolic balance is severely thrown off by normal glucose levels, which exacerbates risk factors for cardiovascular disease.

In addition to these endogenous sources of abnormality, the process of glucose metabolism, and exposure to external substances, such as those found in advanced glycation end products (AGEs), may be amplified by factors in nutrition as well as in the environment, leading to the activation of proatherogenic processes. Although a plethora of research has exposed the deleterious effects of glucose on extra and intracellular characteristics, their long-term unfavorable effects, such as on glycation and epigenetic variables and metabolic memory [75,76], have also been suggested to play crucial roles in CVD in diabetes mellitus. Moreover, diabetes mellitus on the disturbance of lipid/lipoprotein metabolic activities, in addition to their unique and independent effects, also interrelate with all these glucose-driven processes. This is because the glycation of lipids and lipoproteins could alter those species’ function and, through receptor for advanced glycation endproducts (RAGE)-dependent mechanisms, may mediate and exacerbate cellular perturbation [76,77]. As a result, diabetes mellitus is associated with an increased risk of immediate and long-term effects triggered by glucose.

As altered gene expression patterns and signaling pathways combine with immune cells, blood vessel cells malfunction, increasing the risk of vascular and cardiovascular disease in patients with certain metabolic abnormalities [26].

### Vascular Complications in Diabetes Mellitus

Vascular abnormalities in diabetes are caused by a state of chronic hyperglycemia [78]. These difficulties can develop in large blood arteries, characterized by diabetic macroangiopathy, and in small blood vessels, characterized by diabetic microangiopathy [78]. Such vascular irregularities are due to the irrevocable glycation of proteins that occurs nonenzymatically, as well as changes in the cellular redox potential. Elevation in oxidative stress and the condition of inflammation lead to the development of endothelial dysfunction and a state of increased hypercoagulability.

**Figure 4:**
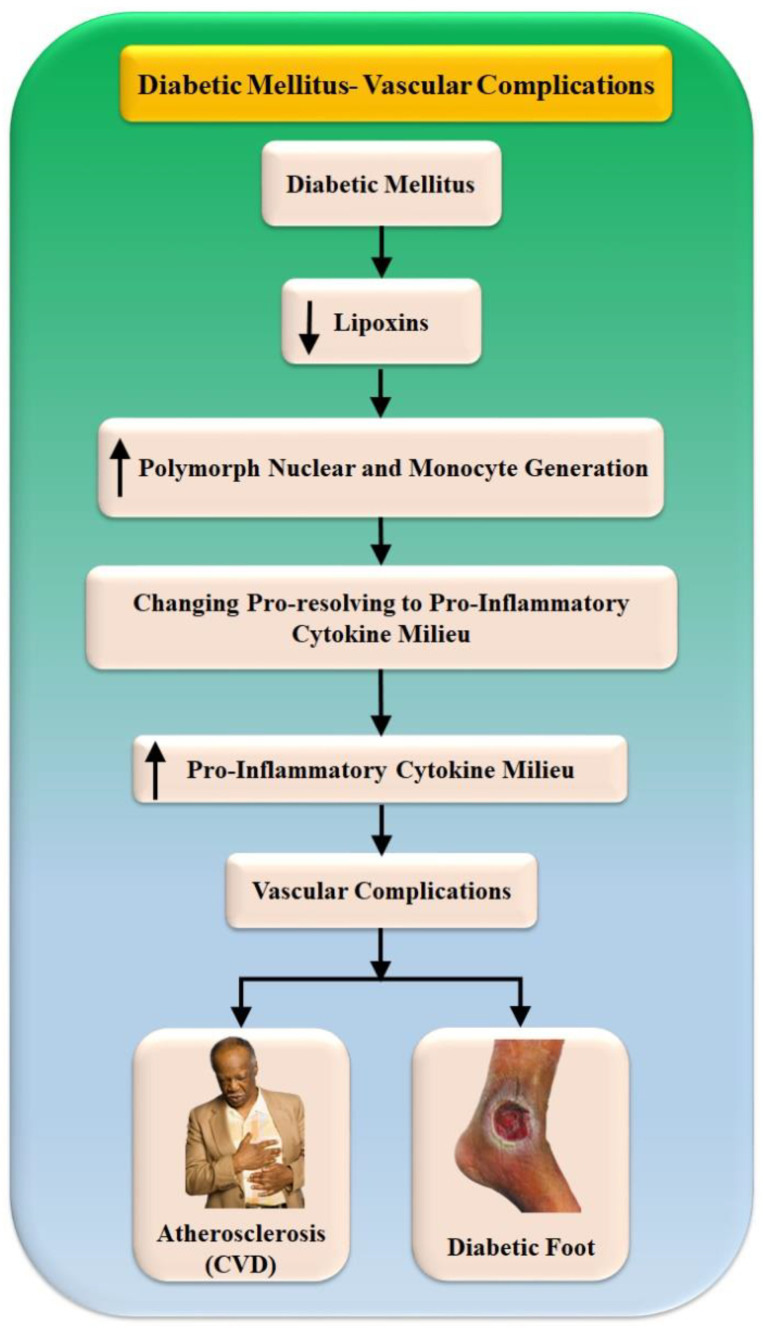
Vascular complications due to diabetes mellitus.

The resolution of inflammation is hampered in diabetic patients, which correlates to the increased levels of TNF-, IL-6, and other proinflammatory cytokines in these patients, as well as to the development and progression of nephropathy and atherosclerosis, and other complications of diabetes [79]. Recent research has demonstrated that proresolving lipid mediators, such as lipoxins, resolvins, and protectins, play a significant role in the resolution of inflammation [22]. These mediators work by suppressing polymorphonuclear and monocyte recruitment and protecting cells from damage, transforming the cytokine environment from proinflammatory to proresolving (Figure 4). As a result, these proresolution lipid mediators have significant therapeutic potential in diabetic renal and cardiovascular disorders [21,80]. The inefficient metabolites of magnification lipid mediators in muscle and adipose tissue contribute to the persistence of chronic inflammation in obesity [81]. This suggests that these lipids could be used to treat insulin resistance, diabetes, and the problems that come with these conditions [82]. Table 1 represents various studies that link DFI and CVD relations.

## 4. ML/DL-Based CVD/Stroke Risk Assessment in Diabetics Foot Ulcer Patients

There is evidence that ML/DL is being used in every industry, including medical imaging [47,88,89]. Deep neural networks (DNNs), a subset of DL, are designed to function like the human brain and have been shown to have several applications [36,90,91,92]. DL makes automatic feature extraction, classification, and segmentation possible via the power of convolution, max-pooling, and various channel maps such as spatial and temporal attention [93,94,95,96]. Multiple publications have detailed the use of AI in the diagnosis and prognosis of CVD [97,98,99] and the forecasting of lesions due to a DFI [51,100,101,102,103,104]. Furthermore, DL has played a crucial role in DFI identification during the presence of comorbidities, including diabetes [105], Parkinson’s disease (PD) [106,107,108,109,110], rheumatoid arthritis [111], and pneumonia [91,112]. In addition to CVD and diabetes,, the presence of such comorbidities in patients profoundly impacts the nonlinear dynamics [113]. As a result, the importance of DL is growing in identifying moderate and high-risk patients with CVD/stroke risk [114,115,116]. Considering this, for superior CVD/stroke risk, an improved set of biomarkers for DFI severity is needed.

Section 4.1 explains the ML/DL-based architecture for evaluating the risk of CVD/stroke in DFI patients. CUSIP quantification using DL which includes the design of wall segmentation using UNet, UNet+, UNet++, and UNet3P, one of the most advanced paradigms, will be discussed in Section 4.2. Furthermore, DL for DFI lesion segmentation and quantification is discussed in Section 4.3. Section 4.4 discussed the challenges in imaging modalities models for CVD risk stratification in DFI patients.

### 4.1. ML/DL-Based Architecture for Evaluating the Risk of CVD/Stroke in DFI Patients

ML techniques were developed for superior segmentation and classification [97,99,114,117,118]. Despite that, it lacked automated feature extraction. In contrast, ML/DL is a powerful framework because it can create automated features by utilizing the underlying knowledge base. It also provides an improved training paradigm in which the nonlinearity between variables and the gold standard can be dynamically adjusted. These two aspects combine to make ML/DL a powerful framework [97,99,114,117,118]. Separating data into training and testing sets is a fundamental tenet of AI algorithms. Our team has already experimented with several DL use cases [119,120,121]. As a result, we arrange our data so that the classes are balanced or if augmentation is needed. Data preparation and the selection of an appropriate cross-validation strategy are two of the most crucial factors to think about before dividing a dataset.

**Figure 5:**
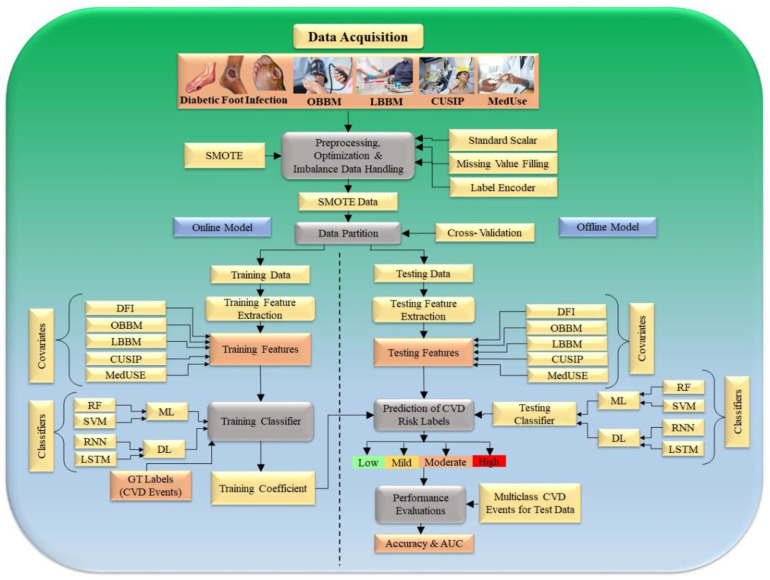
Hybrid model to predict the severity of CVD/Stroke in DFI framework (Courtesy of AtheroPoint™, Roseville, CA, USA permission granted).

The first step, “data preparation or preprocessing”, works in tandem with the second step, “data partition”. Step three generates offline training using training data, and step four estimates the risk of coronary artery disease or cardiovascular disease on the test data (see Figure 5). Two basic procedures make up data preparation or preprocessing: (i) normalizing the data using a typical scalar paradigm that translates the features (risk factors) between 0 and 1, and (ii) augmenting the data using a SMOTE model [95,96]. It has been seen that several algorithms use “PCA-based pooling” which is an established unstructured statistical attribute selection technique as part of the data preparation in the ML area and has been well adapted by our group [34,122].

The second step of the system is responsible for data partitioning; here, the training and testing sets are created with a K10 cross-validation methodology that uses 90% training and 10% testing data. The third step of the architecture is a model generator, where risk variables and the CAS serve as inputs to deep learning classifiers, such as recurrent neural network (RNN) and long short-term memory (LSTM), which generate the offline coefficients. Part four is a prediction paradigm, where the produced model is used to change the test datasets to predict the CAD risk. Keep in mind that the CV is a multimodal paradigm, thus, we will get the predicted CAD value for all the 10 combinations in a cyclic sequence, making sure that no two combinations overlap and that no test data are included in the training set [99,123,124].

One important thing to remember is that the learning algorithm’s embedded feature optimization is a prerequisite [99,125]. The online system is enhanced with a performance component, which calculates accuracy considering the known reference values for the test dataset. The right side below also shows the performance evaluation should the cohort be used using cross-validation protocol, which consists of the computing accuracy, sensitivity, specificity, precision, recall, and *p*-value as conducted in several of our applications [34,39,122]. Table 2 represents various studies used for DFI and CVD prediction. The predictive output labels are either heart failure (cardiovascular events) or stroke (cerebrovascular events) and can be categorized into four parts, such as low, mild, moderate, and high. [126].

#### 4.1.1. CVD Risk Stratification Using ML-Based Classifiers

**Figure 6:**
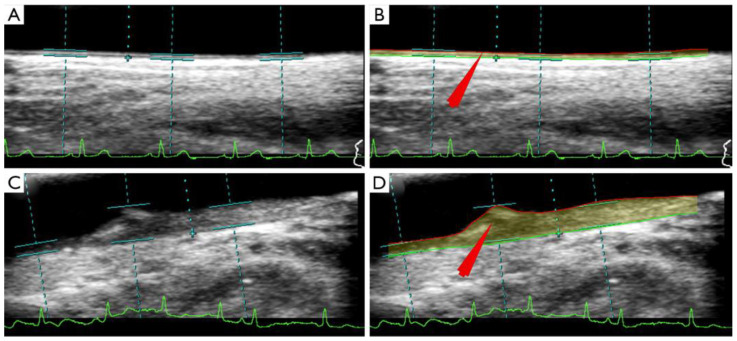
CVD risk stratification is based on an automated AtheroRisk-ML Integrated system. Row 1 (**A**,**B**) is low risk, and Row 2 (**C**,**D**) is High Risk [137].

An ML-based classifier’s purpose is to sort the data it receives into one of several predetermined categories or labels [133]. In the case of a task involving the prediction of CVD or stroke events, for instance, applying the input features to the trained classifier results in a prediction of either the “event” or “no-event” category. The ML-based classifier in this work assigns each patient to either the low-risk or high-risk category, depending on which risk profile they fit into. Meanwhile, we mentioned the fact that the purpose of this study was to devise an ML system that was both effective and economical; therefore, an RF classifier was included in the ML system to perform the risk stratification on the patients [134]. Various studies effectively show the ML-based plaque risk stratification using a Random Forest (RF) classifier. Jamithkar et al proposed (shown in Figure 6) an RF-based ML algorithm that, compared to other ML-based algorithms, has been shown to have a higher predictive capacity [135,136]. As a result, the RF classifier was chosen for the risk stratification of the patients [137].

#### 4.1.2. CVD Risk Stratification Using DL Classifiers

**Figure 7:**
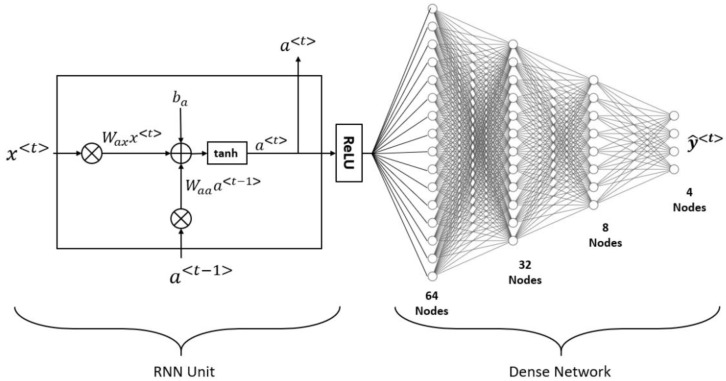
The overall architecture for RNN.

*Recurrent Neural Network (RNN) Classifier*: A study by Rumelhart et al. [138] explained the concept of a subtype of neural network known as an RNN. Using RNNs to approximate nonlinear unknown dynamical systems is a robust architecture [139,140]. Two of the biggest difficulties in training an RNN are the vanishing gradients problem, which has a direct influence on the stability of the model, and (ii) the difficult optimization target [141]. Figure 7 depicts the suggested hybrid design, which consists of a single RNN unit activated with ReLU and four dense layers layered on top of it. There are 64, 32, and 8 nodes, respectively, in the ReLU-activated intermediate dense layers. There are four softmax-activated nodes in the output layer. A complete model is trained to determine a patient’s atherosclerotic risk category based on their input characteristics. Training the model occurred with the help of the loss function categorical cross-entropy loss (CEL) and the optimizer Adaptive Moment Estimation (ADAM). Figure 7 provides a high-level view of an RNN’s structure.

**Figure 8:**
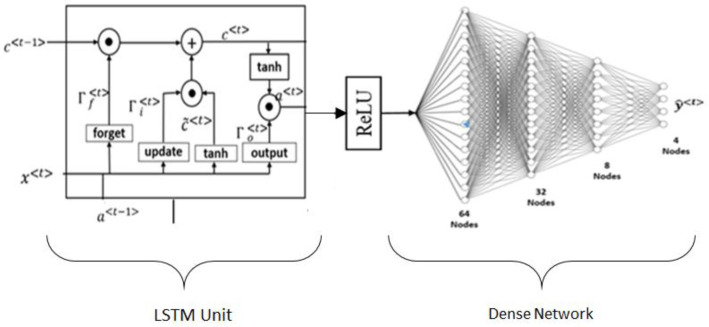
The basic model of LSTM architecture.

*LSTM classifier:* Long-term short memory (LSTM) is one of the types of DL algorithms that can be used to predict the likelihood of developing CVD or a stroke [96]. The issue of long-term dependency is specifically designed to create an LSTM as shown in Figure 8. They do not have to put in a lot of effort to learn how to remember things for extended periods because it is nearly part of their routine. The structure of an RNN always takes the form of a series of modules of the neural network that are repeated. In basic RNNs, this repeating module would frequently produce the same results as a single tanh layer. One of the most important characteristics of an LSTM is its capacity to perform analysis on multiple varieties of data points, such as a single observation. This design incorporates four primary elements, namely, cells, update gates, output gates, and null gates. The design is based on a single component called a cell. The values are stored in the cell at random intervals, and the flow of information or features into and out of the cell is controlled by three gates [142,143,144]. The LSTM consists of four fully connected layers that are fully coupled to one another and stacked on top of one another. When it comes to creating long-term linkages in data, an LSTM performs better than other methods [145].

The dropout strategy is difficult to implement, which makes it difficult to prevent overfitting in LSTMs, which is a common problem with these models. Dropout is a regularization method that works by leaving out the input and recurrent links to LSTM units during the activation and weight-updating steps of training a network. The behavior of an LSTM after being subjected to a variety of random weight initializations is, as a result, quite comparable to that of a feed-forward neural network. Instead, they chose initialization with a small amount of weight [96].

### 4.2. CUSIP Quantification Using UNet Architectures: UNet, UNet+, UNet++, UNet3P

**Figure 9:**
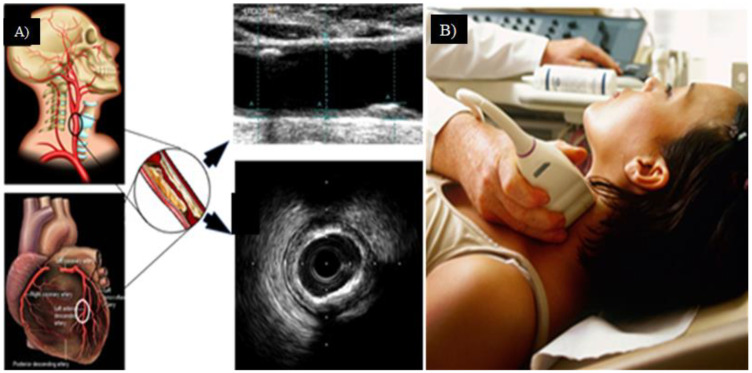
(**A**) CTAD is a potential surrogate marker for COAD, shown using an IVUS-based vascular cross-sectional scan. (**B**) B-mode carotid longitudinal imaging system using linear ultrasound [159].

Since the morphology of the plaque has variability, one needs out-of-the-box techniques which use knowledge-based systems for CUSIP measurements [31]. Such knowledge-based systems evolve a training program that can undergo nonlinear adjustment, as was previously demonstrated in the context of CVD risk stratification [97,98,137,146,147]. The image-based phenotypes that are generated from carotid ultrasound scans are regarded to be CUSIP [67,148]. These phenotypes include total plaque area, average and maximum carotid intima-media thickness (cIMT), intima-media thickness variability (IMTV), geometric total plaque area (gTPA), morphological total plaque area (mTPA), and lumen diameter (LD) [149,150,151] (AtheroEdge™ 3.0, AtheroPoint™, Roseville, CA, USA). This CUSIP is then used to improve the ML algorithm results shown in Figure 9. The segmentation of the carotid wall is helpful in the process of identifying the presence of plaque buildup [152,153,154]. The GT is an important component in the design of ML-based CVD risk stratification. This GT can be a CAD indicator, such as a CT score derived from the CT imaging. CT scoring can also be estimated using a DL framework or one can use plaque tissue characterization using optical coherence tomography (OCT) [155]. The paper by Suri et al. [156] discusses the CT-based scoring system. One can also use an IVUS-based solution for detecting CAD lesions [33,157,158].

**Figure 10:**
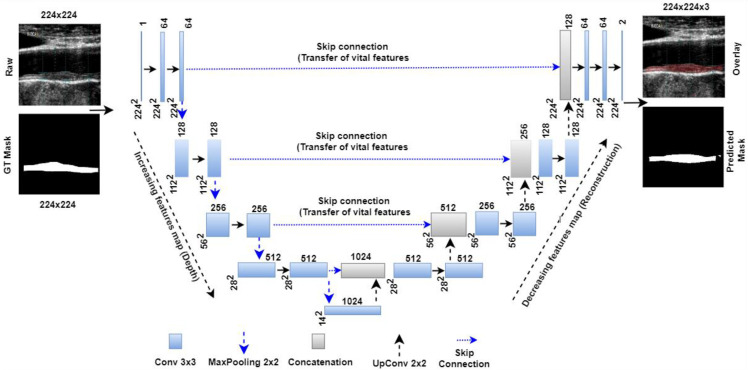
UNet model for segmentation of the wall of an atherosclerotic plaque [121].

Jain et al. [121] have proposed the UNet model for the segmentation of atherosclerotic plaque as shown in Figure 10. The model represents a four-layer DL-based UNet design consisting of four encoders and four decoders on each side of the U-shaped network. The encoder takes down samples while the decoder takes up samples. Each UNet encoder stage has a 2D-convolution, ReLU, and MaxPooling layer. Each decoder stage includes a stack of up-convolution-2D, depth-concatenation, 2D-convolution, ReLU, and MaxPooling layers. Encoder stage one receives a 224 × 224 grayscale US carotid scan. Stage one had 64 convolution filters, and each subsequent stage doubled that number. Each stage has 128, 256, and 512 filters. Each decoder stage halves the number of filters, such as 512, 256, 128, and 64, which are the bottom numerals in the illustration. The bridge network connects the encoder and decoder units. The bridge network has 3 × 1024 filters. Bridge network features can be concatenated to the last encoder stage after downsampling from the first upsampling level. Each encoder stage’s spatial features are sent to the decoder through a skip connection. These functionalities are added to the decoder or bridge network layers. After the final decoder step, the plaque region and backdrop are identified using the softmax classifier layer (pink). An ADAM optimizer reduced plaque segmentation cross-entropy loss.

Deep learning has been improved by the addition of two models that operate independently of each other, a technique known as hybrid deep learning (HDL) [32,160,161,162]. As a result, an SDL-based UNet architecture can be used to create an HDL-based UNet, which may result in improved performance. In addition, given the arrangement of the convolution layer configuration, one can leverage the parallelization notion to increase the HDL designs’ overall performance. The UNet advanced algorithms, such as UNet++ and UNet3P, are shown in Appendix A.

**Figure 11:**
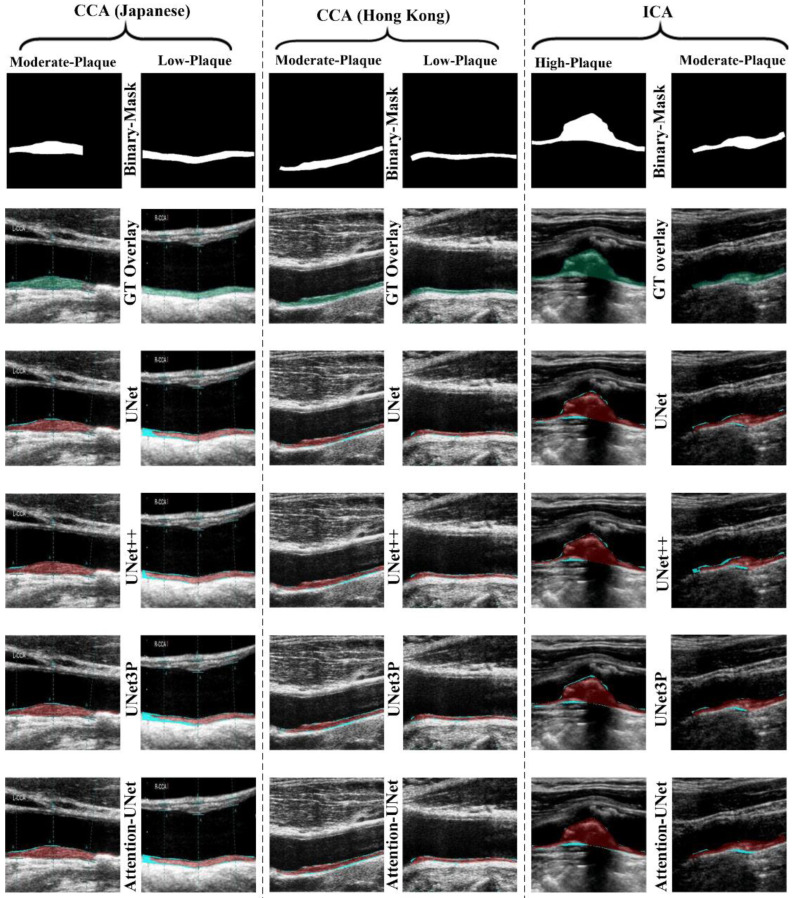
Visualizations of the Japanese, Hong Kong, and United Kingdom (ICA) databases were segmented using UNet, UNet++, UNet3P, and Attention-UNet models [96].

Jain et al. [121] show the role of UNet on two sets of carotid artery scans taken from Japanese and Hong Kong databases and in an unseen AI framework, which allows training on dataset A and testing on dataset B. The UNet model was trained on 330 Japanese DB photos and then evaluated on 300 Hong Kong DB images in the first experiment, referred to as “Unseen AI-1 (Tr: JAP, Te: HK)” [96]. Figure 11 shows the visualization of the carotid data. The UNet training model’s *nine* classification parameters considered were as follows: (i) the reliability coefficient (CC); (ii) the area under the curve (AUC); (iii) the accuracy; (iv) the sensitivity; (v) the specificity; (vi) the precision; (vii) Mathew’s correlation coefficient; (viii) the dice similarity coefficient (DSC); and (ix) the Jaccard index (JI). The mean values of the *nine* classification parameters for the 300 images in the HK DB are 0.8, 0.87, 98.55, 95.41, 98.64, 67.82, 79.29, 78.38, and 65.42 [121].

### 4.3. Deep Learning for Diabetic Foot Ulcer Lesion Segmentation and Its Quantification

Multiple investigations utilizing a variety of imaging techniques have demonstrated DL’s effectiveness in detecting DFI lesions [163,164,165]. In reality, DL has been tried out for lesion detection in several different settings, including (i) the common carotid artery [111,119,166], (ii) the coronary artery [33,167,168], (iii) the brain tumor [169,170,171], (iv) skin cancer [43,122], and (v) CT-based pulmonary imaging [172,173]. The DFI typically has amorphous shapes and permeable boundaries. The skin around a DFI might seem different at different phases, such as redness to callus formation, blistering, granulation, sloughing, bleeding, and scaly skin [174]. The skin around a DFI is crucial because it reveals whether or not the DFI is healing, and it is also a potential extension area [175,176]. Ischemia, inflammation, aberrant pressure, maceration from exudates, and other conditions all raise the likelihood of fragile skin. Similarly, if the skin around the DFI looks healthy, the wound is healing well. The medical imaging of diabetes-related foot ulcers remains complicated [164]. For the representation, we use a smartphone-captured foot image for the modality. However, CT/MRI/Xray images can be used for the imaging modality of foot ulcers [100].

**Figure 12:**
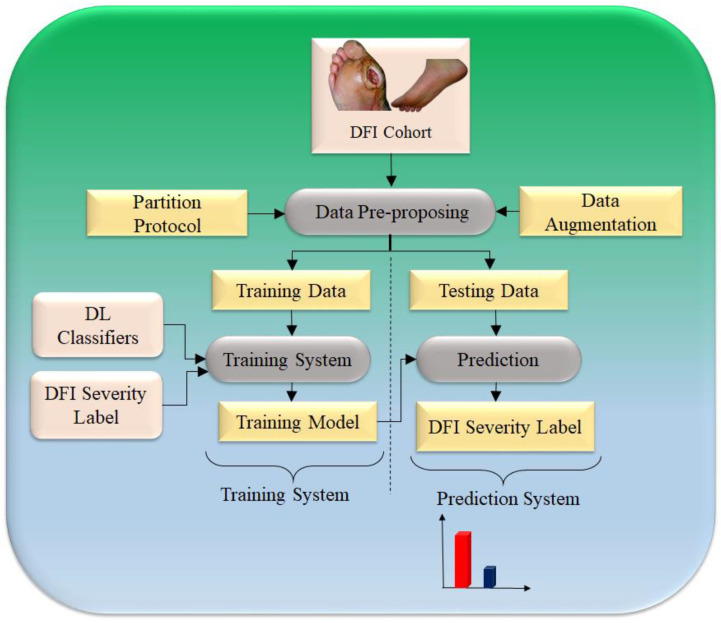
CNN-based model for DFI predication [51].

To improve the process of extracting significant features that are connected to the classification of a DFI, a novel model of a deep CNN-based architecture has been proposed by Alzubaidi et al. [51]. The Directed Acyclic Graph (DAG) principle served as the inspiration for its structure during the design process. When employing these kinds of networks, two major concerns must be addressed. For certain uses, a network that consists of a limited number of different layers and has a straightforward structure is adequate. Furthermore, DFI categorization requires a network that has a more intricate structure to retrieve more information to differentiate between typical and abnormal classes. This not only contributes to an increase in the number of details that can be learned but also to an improvement in the correctness of that learning. Figure 12 illustrates the overall process that our classification follows.

The color, consistency, and discharge of the surrounding skin are all analyzed, and the area is palpated for signs of warmth, swelling, and soreness. Inflammation, usually caused by a wound infection, is indicated by the presence of redness. Black discoloration may indicate ischemia. If something is white and wet, it is because of maceration, but if it is white and dry, it is usually because of increased pressure. Understanding that skin tones affect how things look is crucial. Sometimes, skin lesions that show up red or brown on white appear black or purple. Darker skin colors may hide even mild cases of redness. The process of segmentation is designed by first extracting texture features and color variables from small patches of wound images, and then using ML algorithms to identify the patches of skin as either normal or aberrant [177,178,179,180].

**Figure 13:**
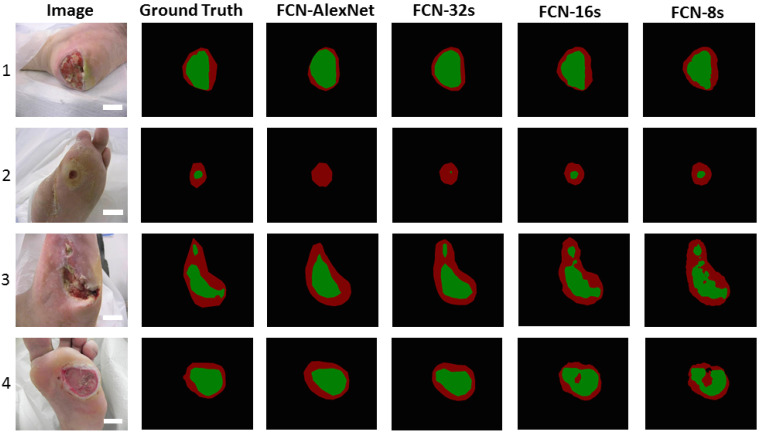
Four different FCN models (columns 3–6) and the gold standard (column 2) demonstrate the segmentation of the DFI area (green) from the skin (red) around it [51].

Here, we focus on an image-based DFI lesion segmentation and its quantification that extracts features (covariates) during the DL paradigm. In DL, manual delineations of DFI lesions are challenging and are also vital for the design of offline DL training models. Figure 13 shows a few instances in which FCN-AlexNet and FCN-32s models can detect the small DFI and distinct surrounding skin or detect a very small part of them. Hyperparameter adjustment during training is a crucial part of DL for achieving optimal system performance. To avoid overfitting and ensure generalization, it is necessary to optimize (i) the learning rate, (ii) the number of epochs, (iii) the batch size, (iv) the normalization of batches, and (v) the addition of dropout layers. As a corollary, the ideal DL architecture necessitates the use of many biomarker sets, each with its unique collection of data, on a big data platform that guarantees a multiresolution platform for speedy implementation [94]. To guarantee faster performance, such pretrained models can benefit from transfer learning when used for DFI lesion segmentation [120,180,181,182,183].

### 4.4. Challenges in CVD Risk Stratification on DFI Patients

Despite the availability of a wide range of diagnostic imaging techniques for the examination of diabetes-related foot problems, it is still difficult to differentiate between neuroarthropathy and osteomyelitis. The early and precise diagnosis of diabetic foot problems can assist in lowering the prevalence of infection-related comorbidities, the requirement for hospitalization, the length of hospitalization, and the prevalence of major limb amputations.

The main procedures that are used at this time for the examination of diabetes-related foot problems include traditional radiography, computerized tomography, nuclear medicine scintigraphy, magnetic resonance imaging, ultrasound, and positron emission tomography [184,185]. On the other hand, each one of these modalities cannot provide enough information by itself; therefore, a multimodal approach is required to arrive at an accurate diagnosis [186].

Therefore, we hypothesize that DL models can execute specific tasks, such as automated disease diagnosis, with more precision and efficiency than ML models, and that they serve as a second level of validation on the diagnosis. Models that have been trained using DL can be used for a broad variety of challenges, such as differential diagnosis, enhancements to image acquisition, and picture-based quantification.

The AI models have some challenges: (i) The data size must be large. If the data size is not big enough, SMOTE should be used during training to make it bigger. (ii) GT should be evaluated correctly for CVD risk, such as CAD in the training model. (iii) Optimization must be performed during the training of the CVD design. (iv) The correct CUSIP should be found by using UNET with attention channel maps. (v) All biomarkers, such as OBBM, LBBM, CUSIP, MedUSE, and DFI Severity, must be collected in the right way. (vi) DFI Severity DL system should give the risk appropriate and be validated by the Diabetologist or even surgeons dealing with foot amputations. (vii) Strong ML or DL models, such as XGBOOST, RNN, and LSTM, must be taken into account. (viii) If the ML models are not strong, one can switch to ML or DL ensemble models.

## 5. Discussion

### 5.1. Principal Findings

This is the first study to investigate the risk factors and gold standards for CVD and stroke in DFI patients based on their symptoms. The findings highlight the importance of selecting CVD and stroke risk-assessment approaches for DFI patients, especially those at high risk for CVD and stroke. Diagnosing a heart issue in a patient with a DFI is aided by surrogate carotid artery imaging. It has become clear from our research that ultrasound-based imaging techniques are the most practical for carotid atherosclerotic imaging. Furthermore, under the DFI framework, AI-based algorithms are the best option for the risk stratification of CVD/stroke.

A DFI is widely considered harmful to the brain and the heart. The review shows how a DFI worsens CVD and stroke in a progressive chain of events. We propose an approach to employing AI to aid in the diagnosis of CVD/stroke risk stratification in the DFI framework. Therefore, we can employ gold standards, such as coronary artery CT scores or coronary IVUS plaque burden, for superior AI training-based design for offline model generation, which can then be used for transforming the test patient features for CVD/stroke risk prediction. Using an AI-based model, we can effectively monitor these patients and prevent any CVD-related adverse long-term effects. Thus, for the DFI framework, ML and DL models can help provide a more precise assessment of the risk of CVD and stroke. The model could be taught so that it operates automatically and quickly. This is a game-changer for modern healthcare systems, particularly in identifying CVD and stroke risks in DFI patients. Clinicians can use the AI models’ vascular and cerebrovascular data-based results to better counsel DFI patients and advise them on their CVD/stroke risk stratification.

### 5.2. Benchmarking

An analysis of the available data reveals that a DFI and CVD have been connected in a few studies using OBBM, LBBM, and MedUSE. In the study, AI’s role in identifying combined CVD/stroke and a DFI has only been briefly mentioned. The AI model is only utilized by selecting a few articles within the DFI framework to describe the severity of CVD.

Parthiban et al. [127] explained the role of classifiers that can be helpful in the early diagnosis of the diabetic patient’s susceptibility to developing heart disease. The patients can then be warned to adjust the way they live as a result. Diabetic individuals will be less likely to develop heart disease, leading to lower mortality rates and, therefore, less overall healthcare costs. An SVMs classifier was explored that used a cross-validation protocol and showed an accuracy of 83.32%. Therefore, the use of this SVM model for the categorization of the diabetic dataset is something that may be advocated.

Jelinek et al. [128] focused on automatically identifying severe diabetic neuropathy using a brand-novel algorithm called Glioblastoma Multiforme (GBML). The study evaluated the specificity and sensitivity of the findings using GBML and compared the results against other ML methods. The patient size was 242. The uses K5 CV protocol. The GBML test for identifying acute diabetic neuropathy reached the highest degree of performance, with a sensitivity of 0.98 and a specificity of 0.89.

Zarkogianni et al. [129] carried out a study into the application of cutting-edge ML methods, the bilinear model, and ensemble learning to produce CVD risk scores for a population with type 2 diabetes. The utilization of a subsampling learning strategy resulted in the production of several primary models based on Hybrid Wavelet Neural Networks (HWNN) and self-organizing maps (SOM). The independently trained primary models’ results were combined using DL and the results were then compared with one another. The models were evaluated using information taken from the medical records of 560 T2DM patients. The best discrimination performance achieved an area under the curve (AUC) of up to 71.48%.

Segar et al. [131] proposed an innovative risk prediction tool, WATCH-DM, which was tested on a well-phenotyped clinical study of patients with type 2 diabetes and cardiovascular disease or risk factors, but no history of heart failure at baseline. It identified patients who face a heart failure risk of up to 20% in the next five years. Since the data needed to calculate the WATCH-DM risk score are collected during the routine clinical care of patients with type 2 diabetes, therefore, integrating the WATCH-DM risk score into electronic health record systems or mobile health applications will provide a powerful tool for clinical practice. The advantage of WATCH-DM is that it does not require a particular cardiovascular biomarker or supplementary imaging examination. More research needs to be done to determine whether or not the WATCH-DM can be effective compared to other therapeutic options that are now accessible, such as sodium-glucose transport proteins (SGLT2i).

Aggarwal et al. [116] demonstrated diabetes mellitus (DM) causes hyperglycemia. Type 1 and type 2 diabetes are insulin-deficiency and insulin-resistance conditions. It can induce atherosclerosis, stroke, and MI. Neurodegeneration and autonomic dysfunction are also present. Autonomic balance regulates nonlinear physiological factors. The data size of 526 was produced from ECG data to evaluate 13 regressive HRV parameters and test ANN. With these inputs, an ANN design (13:7:1), at a 0.01 learning rate, achieved 86.3% classification accuracy. SVM differentiated diabetic and controlled individuals with an accuracy of 90.5%. Nonlinear HRV parameters reveal different changes owing to diabetes, so they can be combined with ML algorithms to construct a noninvasive, low-cost real-time diabetes prognosis system.

Derevitskii et al. [115] proposed that DM is among the most frequent forms of diabetes, also known as chronic diabetes. This particular form of diabetes is among the healthcare industry’s most pressing concerns today. This disease is linked to several other conditions that simultaneously raise the risk of CVD and premature impairment. Patients diagnosed with type 2 diabetes have an elevated risk of various problems. In the case of patients such as these, medical doctors required methods that were more realistic for estimating the potential for future difficulties.

Karhu et al. [86] explained that the role of diabetes is extremely common in individuals who have already been diagnosed with CVD or chronic heart failure, and it is associated with a large increase in the likelihood of unfavorable outcomes. However, the persistently poor outcomes of people with diabetes mellitus highlight the importance of diabetes-specific systematic reviews and novel therapeutics aimed at specific pathophysiological requirements such as diabetic vascular and heart disease.

Schuett et al. [87] proposed that diabetes is prevalent in individuals who have already been diagnosed with CVD or chronic heart failure. It is essential to provide holistic care that focuses on lowering overall cardiovascular risk by employing various prevention methods to significantly cut the risk of cardiovascular events, progress to CHF, and mortality. However, the continually poor results of individuals with DM emphasize the importance of a diabetes-specific systematic review. Innovative therapeutics for particular pathophysiological conditions require an assessment of diabetic vascular and heart disease. To the best of our knowledge, no AI study has ever been able to provide us with information that is both clear and helpful regarding the CVD and stroke risk classification of DFI patients. The benchmarking analysis for the studies listed in Table 3 is presented below.

### 5.3. Special Note on Casual Relationship between DFI and CVD

DFIs are vascular complications of diabetes mellitus associated with high mortality and morbidity. A few authors discovered a higher prevalence of major, previous, and new-onset cardiovascular and cerebrovascular events in diabetic patients with foot ulcers than in those without these complications [23,52,187,188]. This is consistent with diabetes’ complicated interplay of factors with inflammatory metabolic diseases and their effects on the cardiovascular system, which could explain the increased morbidity and mortality levels in diabetic patients with amputations [189]. Inflammatory markers, such as IL-6 plasma levels and resisting, in diabetic participants validated the pathogenic issue of the “adipovascular” axis, which may add to the cardiovascular risk in type 2 diabetics. This “adipovascular axis” could be linked to the cause of foot ulcers in people with diabetes through microvascular and inflammatory mechanisms [2].

### 5.4. A Short Note on the Effect of COVID-19 on DFI Patients

COVID-19 has been shown to have affected several organs of the human body, such as the brain and heart [190]. A DFI causes more disability and death than any other diabetes condition. DFIs that do not heal despite treatment are the primary cause of hospitalization, amputation, disability, and mortality among people with diabetes [191]. People with diabetes, especially those with extensive foot ulcers, present significant issues in the face of a global pandemic such as COVID-19 [192]. To face the COVID-19 outbreak, the traditional diabetic foot treatment routine is no longer appropriate. Various studies have commented on a novel procedure for treating a patient with a DFI in the setting of the worldwide COVID-19 pandemic [188,193,194]. DFIs were classified as (i) mild (having no wound or tiny wound, no infection, and stable condition), (ii) moderate (having complex and refractory infection wound), or (iii) severe (having dry gangrene, sore in the injury, body temperature, and sepsis symptoms) [195]. Patients with generalized diabetic foot issues can receive treatment at home with the help of telemedicine. This allows clinicians to instruct patients and encourage them to do a selfexamination of the foot, how to change wound dressings, and administer medications [192]. Patients with severe problems are referred to the hospital’s outpatient clinic for treatment following a positive COVID-19 screening. Patients with a severe DFI who have been diagnosed or suspect that they have a COVID-19 infection require immediate isolation and ongoing quarantine. Patients with a low or mild DFI will be discharged to continue their care at home under telemedicine monitoring and physician supervision, while patients with a critical DFI will be admitted to the hospital following a COVID-19 screening [196]. During their hospital stay, patients with a DFI in a serious condition will receive a variety of treatments, ranging from rest and medication to debridement and local dilatation, and even amputation [197].

### 5.5. A Short Note on Bias in Deep Learning Systems for CVD/Stroke Risk, DFI, CUSIP Measurements

Bias was unnoticed in early computer-aided diagnosis systems [198]. Recently, the role of bias estimation in AI models has quickly emerged. Several factors are important, such as the sample size used in the training model design step of the DL algorithms, which is very important to consider. Furthermore, there is bias in AI due to several factors, including (i) a lack of clinical testing of AI techniques, (ii) scientific validation, (iii) failing to meet the gold standard, (iv) comorbidities, (v) a lack of big data configuration, (vi) failing to perceive the proper disease severity ratio, and (vii) variabilities in CVD [199]. As a consequence of this, when DFI-associated CVD symptoms (or risk variables) are investigated as inputs to an AI model, it is essential that the AI model be stable, accurate, and have a small amount of AI bias [152,156,173,200,201]. It is possible to observe that the database contains patient characteristics that are particular to a given region. Because of this, the model can produce false positive or negative results for other places, which would make the algorithm biased [185,202].

### 5.6. Work Flow for CVD Risk Stratification for DFI Patients

**Figure 14:**
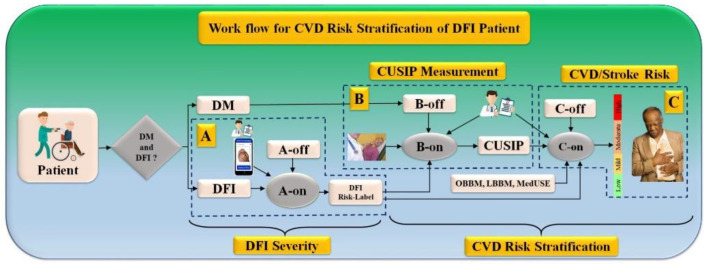
The overall architecture of CVD screening on DM and DFI patients. **A-on**: Online DL-based FDI severity system, **A-off**: Offline DL-based FDI severity system, **B-on**: Online DL-based Carotid wall quantification system, **B-off**: Offline DL-based Carotid wall quantification system, **C-on**: Online ML-based CVD Risk Assessment system, **C-off**: Offline ML-based CVD risk assessment system, DM: Diabetes Mellitus, DFI: Diabetic foot infection, CUISP: Carotid ultrasound image phenotype.

The workflow of the CVD/stroke risk stratification of DFI/DM patients can be seen in Figure 14. The pipeline consists of three major systems, labeled A, B, and C. System A consists of a DFI severity estimation given the patient’s condition if the patient has a DFI. This DFI is an online system called **A-on**. System B consists of the CUSIP measurements which is also an online AI-based system, called **B-on**. The final system C is also an online system, such as a machine or deep learning system, for CVD/stroke risk stratification labeled as **C-on**. Note that all three online AI-based systems are supervised and, hence, must be executed by the trained offline systems called **A-off**, **B-off**, **and C-off**. Note that the **A-on** system accepts real camera phone images of the DFI whose DFI severity needs to be estimated using the **A-off** system. The output of the **A-on** system is the DFI severity. The **B-on** system accepts the surrogate imaging of CAD, so-called carotid imaging, along with the **B-off** trained system leading to the CUSIP measurements. Finally, the **C-on** system is triggered by taking the inputs of online laboratory-based biomarkers, such as LBBM, OBBM, CUSIP, MedUSE, and DFI-severity, and the **C-off** trained system to estimate the CVD/stroke risk stratified system.

The main feature of the model is cost-effectiveness. The imaging device used for diabetic foot infection image capturing is a smartphone. CUSIP is used for the carotid artery scan. There is no necessity for extra devices.

### 5.7. Strengths, Weakness, and Extensions

The presented research article explains the various essential aspects of risk stratification for CVD and stroke patients with a DFI disease. Because of its improved nonlinear adjustment between the variables and the gold standard, DL provides better training and more accurate risk prediction. Additionally, the system gives it thorough predictors, such as OBBM, LBBM, CUSIP, MedUSE, and DFI as covariates, in addition to providing an estimation of the lesion size based on the wound scans of the diabetic foot. The role of an LSTM or RNN, an extremely effective strategy for creating the DL system for predicting the risk of CVD and stroke, was given. In conclusion, the DL system is generalized, and this generalization can be changed by including additional covariates and comorbidities, such as diabetes, rheumatoid arthritis, renal disease, coronary artery disease, etc.

While DL brings some benefits to the system, one must always ensure that the system is optimized to take advantage of these benefits. In addition, the DL system needs a solid gold standard for (a) lesion annotations and (b) CVD/stroke gold standard collection in cohorts. Both of these steps take a significant amount of time, and they also have associated costs. Last, but not least, as was said before, deep learning systems are vulnerable to artificial intelligence bias because of their overperformance in terms of accuracy and lack of interpretability.

When it comes to the design of extensions, ensemble-based methodologies allow for the creation of superior DL systems. Big data are an option that could be considered to strengthen the DL system by using a larger sample size and more data sources. If only a few participants are in the cohort, the DL system can be improved by incorporating augmentation designs. One can also integrate the conventional image-processing models with advanced DL models for superior feature extraction [5]. Furthermore, as part of the extension, one can learn about ulcers using multimodality imaging [203]. Another important component is to monitor the CVD/stroke risk with the changing DFI lesions. This can incorporate tools for image registration [204]. Last, but not least, the DL system needs to be updated with the latest round of pruning so that smaller training storage models [205] and evolutionary approaches [206] can be used.

## 6. Conclusions

This in-depth study brought to light the significance of CVD and stroke risk predictions for people with a DFI living in a diabetic environment. Additionally, we demonstrated how a DFI combined with hypertension can lead to strokes in both the vascular and cerebral systems. This review focused on how a DFI may contribute to the already complex nature of CVD and stroke. Therefore, it is essential to classify DFI patients’ risk of CVD and stroke. Carotid screening is a noninvasive, reduced alternative to traditional imaging that can be used to monitor people with a DFI for CVD and stroke. The low-cost B-mode ultrasonography will also help to describe the plaque tissue in patients with a DFI, which can improve the estimation of the risk of CVD and stroke. The severity of the DFI can be diagnosed and quantified using wound scan pictures of foot lesions. This information can then be used as a covariate in the DL design process.

An artificial intelligence-based model for predicting the risk of CVD and stroke in DFI patients was described using the AI framework. Because of this, we have discussed the function of an AI-based model that, based on the DFI risk profile of the patient, can reliably categorize patients diagnosed into risk groups for CVD and stroke. Finally, we explore the function that AI plays in this setting as well as the engagement of a DFI in the CVD/stroke paradigm.

## Figures and Tables

**Table 1:** Relationship between the diabetic foot, diabetic syndrome, and cardiovascular disease.

SN	Citations	Relationship	ME	PS	OUTCOME	TRE
1	Feleke et al. [28] (2007)	DFI and CVD	LBBM, OBBM	2818	DFI Infections led to morbidity, with the combined effect of CVD leading to mortality. Following diabetic foot ulcers came TB, skin and subcutaneous infections, and pneumonia.	NR
2	Brownrigg et al. [14] (2012)	DFI with CVD risk of mortality	LBBM	3619	DFI patients have a higher risk of all-cause mortality than other diabetics. CVD contributes to this risk.	NR
3	Matheus et al. [83] (2013)	Diabetes and CVD	LBBM	NR	Diabetes prevention is the most effective way to lower CVD risk. Traditional, changeable heart disease risk factors are still essential for diabetes people.	NR
4	Tuttolomondo et al. [16] (2015)	DFS as a Cardiovascular Marker	LBBM	NR	In addition to peripheral sensory neuropathy, deformity, and trauma, other risk factors, including calluses, edema, and peripheral vascular disease, have been identified as etiological contributors to the formation of diabetic foot ulcers.	NR
5	Domingueti et al. [13] (2015)	Diabetes and CVD	LBBM	NR	Vascular problems in type 1 and type 2 diabetes are closely linked to endothelial dysfunction, hypercoagulability, inflammation, and the poor resolution of inflammation.	NR
6	Al-Rubeaan et al. [27] (2015)	DFI and CVD	LBBM	NR	Neuropathy and PVD are major risk factors for diabetic foot problems. Diabetic retinopathy is a major independent risk factor for diabetic foot issues. CVD risk factors are common among diabetics, and primary and secondary prevention strategies are essential to reduce morbidity and expense from this chronic condition.	NR
7	Bertoluci et al. [11] (2017)	Diabetes and CVD	LBBM	NR	CVD risk is increased 2- to 4-fold in people with type 2 diabetes, however, due to the disease’s extreme variability, the two conditions cannot be regarded as risk equivalents. To tailor care to each patient, risk assessment is essential.	NR
8	Dietrich et al. [15] (2017)	DFI as a Predictor of CVD and Mortality	LBBM	NR	DFS is linked to CVD and death. DFI’s connection with renal failure and retinopathy indicates the evolution of micro- and macrovasculopathy, neuropathy, chronic inflammation, and lipotoxicity.	NR
9	Mishra et al. [24] (2017)	DFI and CVD	LBBM	NR	Patients diagnosed with DFI have an increased risk of death from any cause compared to other diabetics. The risk is increased by cardiovascular disease.	NR
SN	Citations	Relationship	ME	PS	OUTCOME	TRE
10	Petrie et al. [84] (2018)	Diabetes and vascular complication	LBBM	NR	Diabetes and hypertension increase the possibility of CVD. Oxidative stress, inflammation, and fibrosis, which cause microvascular and macrovascular problems of diabetes, also cause vascular modification.	NR
11	Serhiyenko et al. [85] (2018)	Cardiac autonomic neuropathy in diabetes	LBBM	NR	CAN is a frequent, undiagnosed consequence of DM that increases CV morbidity and mortality. As cardiac denervation could be prevented and partially reversed in early disease stages, DM patients should be screened for it.	Yes
12	Shariful et al. [12] (2020)	Diabetes and CVD	LBBM	1262	Diabetes increased CVD risk at an early age. To reduce future CVD risks, diabetics must reduce cigarette usage and improve BP control.	NR
13	Balasubramanian et al. [20] (2021)	DFI and Microcirculation	LBBM	NR	Microcirculation plays a crucial function in tissue injury and inflammation homeostasis and resistance. Furthermore, the latest evidence supports the disruption of microcirculation as the weak link in the sequence of events that leads to DFI.	NR
14	Karhu et al. [86] (2022)	Diabetes and CVD	LBBM	2535	Intermittent hypoxia is worse in people with preexisting CVD, and diabetes and CVD accelerate IH deterioration. Intermittent hypoxia is a pathophysiological hallmark of sleep anemia that increases the risk for severe health consequences. Patients with diabetes or CVD should receive additional attention for sleep anemia screening and follow-up monitoring.	NR
15	Schuett et al. [87] (2022)	Diabetes and CVD	LBBM	NR	Diabetes and hypertension trigger CVD. Oxidative stress, inflammation, and fibrosis promote microvascular and macrovascular diabetic complications.	NR
16	Qiu et al. [57] (2022)	DFI and CVD	LBBM	423	The development of a diabetic foot ulcer was associated with a considerably greater death risk from all causes as well as from cardiovascular disease compared to that of a control group of those who had diabetes mellitus but did not have DFI.	NR

SN: serial number, RELATION Diabetic Foot and CVD, ME: method of evaluation, PS: patient size, OE: outcome, TRE: Treatment, NR: not reported, CVD: Cardiovascular disease, DFI: Diabetic Foot Ulcer, DFS: Diabetic Foot Syndrome, DM: Diabetic Mellitus, CAN: Cardio Autonomic Neuropathy, LB: Lab-base, OB-Office base, TB: Tuberculosis, PAD: Peripheral Arterial Disease.

**Table 2:** Studies show the role of AI in the diagnosis, and prediction of, DM, DFI, and CVD.

SN	Citations	IC	DS	REL	PRE	ClassTy	TOC	ML/DL	ACC %	AUC	SEN	SPE	F1	MCC
1	Parthiban et al. [127] (2012)	LBBM	341	DM, CVD, and AI	CVD	SVM	NB	ML	74.23	0.73	0.79	NR	NR	NR
2	Jelinek et al. [128] (2016)	OBBM, LBBM	88	DM, CVD, and AI	CVD	SVM	RF	ML	81.00	0.89	0.91	0.89	NR	NR
3	Zarkogianni et al. [129] (2017)	OBBM, LBBM	560	DM, CVD, and AI	CVD	SVM	NB	ML	76.34	0.87	0.79	0.76	NR	NR
4	Basu et al. [130] (2018)	OBBM, LBBM	2529	DM, CVD, and AI	Death	PCA	KNN, DT	ML	84.34	0.843	0.87	NR	0.76	0.843
5	Dinh et al. [101] (2019)	OBBM, LBBM	131	DM, CVD, and AI	DM, CVD	XGBoost	RF	ML	84.10	0.81	0.78	0.73	NR	NR
6	Segar et al. [131] (2019)	OBBM, LBBM	319	DM, CVD, and AI	Heart Failure	LDA	RF	ML	76.00	0.778	0.76	NR	0.79	0.778
7	Aggarwal et al. [116] (2020)	OBBM, LBBM	526	DM, CVD, and AI	CVD	SVM	ANN	ML	86.00	0.863	NR	0.81	0.71	NR
8	Derevitskii et al. [115] (2020)	OBBM, LBBM	8139	DM, CVD, and AI	Stroke, DM	XGBoost	NB	ML	84.53	0.87	0.91	0.86	NR	NR
10	Hossain et al. [132] (2021)	OBBM, LBBM	4819	DM, CVD, and AI	CVD	SVM	RF	ML	88.16	0.80	NR	NR	0.88	NR
11	Longato et al. [103] (2021)	OBBM, LBBM	24676	DM, CVD, and AI	CVD	SVM	CNN	DL	79.81	0.76	0.84	NR	0.79	NR
SN	Citations	IC	DS	REL	PRE	ClassTy	TOC	ML/DL	ACC %	AUC	SEN	SPE	F1	MCC
13	Hyerim et al. [102] (2022)	OBBM, LBBM	10442	DM, CVD, and AI	DM, CVD	LR, DT	CNN	DL	80.88	0.86	0.81	NR	NR	NR
14	Goyal et al. [30] (2020)	OBBM, LBBM	7136	DFI and AI	Diabetic foot Infection	NR	CNN	DL	91.21	0.93	0.84	0.89	NR	NR
15	Alzubaidi et al. [51] (2020)	OBBM, LBBM	754	DFI and AI	DFI	KNN	DNN	DL	93.04	0.91	0.87	0.83	0.94	NR
16	Khandekar et al. [100] (2021)	LBBM (IR)	202	DFI and AI	Diabetic foot	6 Models	CNN	DL	92.51	0.92	NR	NR	0.81	NR
17	Isaza et al. [29] (2021)	OBBM, LBBM	146	DFI, CVD, and AI	DFI	PCA	CNN	DL	88.24	0.84	0.86	0.79	NR	NR

SN: serial number, IC: input covariates, DS: data size, REL: Relation, PRE: Prediction, ClassTy: Classifier type, OBBM: Office base biomarker, LBBM: Lab base biomarker, FE: feature extraction, TOC: Type of classifier, ACC: Percentage accuracy, SEN: Sensitivity, SPE: Specificity, MCC: Mathew coefficient correlation, AUC: Area under curve, DL: Deep learning, ML: Machine Learning, CNN: Convolution neural network, DFI: Diabetic Foot Infection, DNN: Deep neural network, RF: Random forest, SVM: Support vector machine, DT: Decision tree, LR: Logistic Regression, US: Ultrasound, NR: not reported.

**Table 3:** Comparing the proposed review against previous reviews on joint DFI and CVD.

SN	Citations	Year	DFI^a^	DM^b^	CVD^c^	DI^d^	WI^e^	AI^f^	RS^g^	ClassTy^h^	ML/DL^j^	ACC %^k^	AUC^l^	SEN^m^	SPE^n^	F1^o^
1	Parthiban et al. [127]	2012	✕	✓	✓	✕	✕	✓	✕	✓	✓	✓	✓	✕	✕	✕
2	Jelinek et al. [128]	2016	✓	✓	✓	✕	✕	✕	✕	✕	✕	✕	✕	✕	✕	✕
3	Zarkogianni et al. [129]	2017	✕	✕	✓	✓	✕	✓	✕	✓	✓	✓	✕	✕	✕	✕
4	Segar et al. [131]	2019	✓	✓	✓	✕	✕	✕	✕	✕	✕	✕	✕	✕	✕	✕
5	Dinh et al. [101]	2019	✓	✓	✓	✓	✓	✓	✓	✓	✓	✓	✕	✕	✕	✕
6	Aggarwal et al. [116]	2020	✓	✕	✕	✓	✕	✓	✕	✓	✓	✓	✓	✕	✕	✕
7	Derevitskii et al. [115]	2020	✓	✓	✓	✕	✕	✕	✕	✕	✕	✕	✕	✕	✕	✕
8	Karhu et al. [86]	2022	✓	✓	✓	✕	✕	✕	✕	✕	✕	✕	✕	✕	✕	✕
9	Schuett et al. [87]	2022	✓	✓	✓	✕	✕	✕	✕	✕	✕	✕	✕	✕	✕	✕
10	Hossain et al. [132]	2021	✓	✓	✓	✕	✓	✓	✕	✓	✓	✓	✓	✕	✕	✕
11	Longato et al. [103]	2021	✓	✓	✓	✕	✕	✓	✕	✓	✓	✓	✓	✕	✕	✕
12	Hyerim et al. [102]	2021	✓	✓	✓	✕	✕	✓	✕	✓	✓	✓	✓	✕	✕	✕
13	Maindarkar et al. (proposed)	2022	✓	✓	✓	✓	✓	✓	✓	✓	✓	✓	✓	✓	✓	✓

DFI^a^: Diabetic foot Infection, DM^b^: Diabetic Melliuties, CVD^c^: Cardiovascular diseases, WI^d^: Wound Imaging, CI^e^: Carotid Imaging AI^f^: Artificial Intelligence, RS^g^: Risk Stratification, ClassTY^h^: Type of Classifier, ACC^k^: Accuracy, AUC^l^: Area under curve, SEN^m^: Sensitivity, SPE^n^: Specificity.

**Figure A1:**
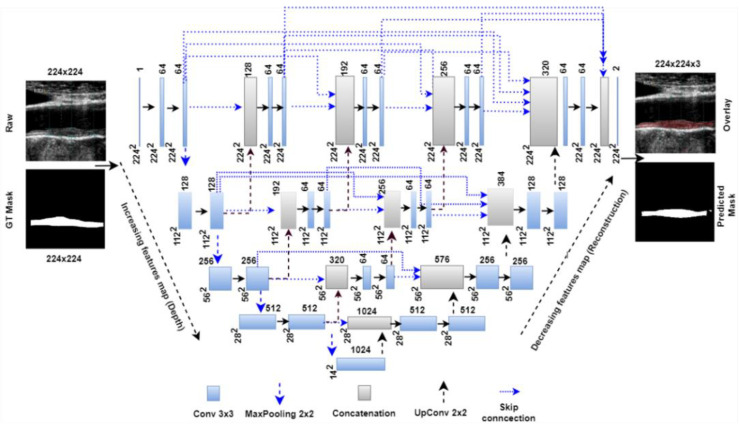
UNet++ Architecture.

**Figure A2:**
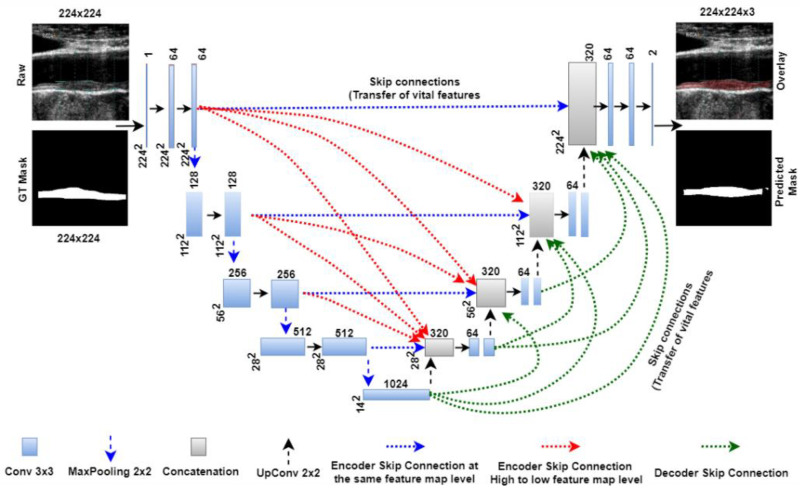
UNet++ Architecture.

## Data Availability

No data availability.

## Appendix A. UNet+ and UNet++, and UNet3P Architecture

The UNet+ and UNet++ designs are depicted below in Figure 10 and Figure A1, respectively [207]. Both of these networks are enhanced variations of the UNet’s architecture. In each of these architectural designs, the links between the encoder and decoder stages are handled by something called a “dense skip network (DSN)”. The UpConv layer is the first in the DSN, which is then proceeded by concatenation and two levels of convolution. The output of the subsequent encoder stage is passed through the UpConv layer and into the concatenation layer, where it is merged with the output of the same encoder level. Both UNet+ and UNet++ have the same quantity of DSNs at every stage of the encoding and decoding process. It is important to note that, in the case of the UNet+ architecture, each DSN is only connected to its previous skip network output, as shown in Figure 10, whereas in the case of the UNet++ architecture, every DSN is linked to all prior DSNs in the same phase via avoiding network outputs, as shown in Figure A1. Figure 10 shows the UNet+ architecture, and Figure A1 shows the UNet++ architecture.

The UNet3P network is yet another iteration of the original UNet protocol. This model presents a novel approach to full-scale skip connection that improves upon the utility of multiscale features. High-level definition of feature maps generated from multiscale features is combined with lower-level specifics of the region of interest to use these full-scale skip connections. A lack of interconnectivity between features on

Different scales are a weakness shared by UNet, UNet+, and UNet++. Therefore, UNet3P takes advantage of the multiscale features by incorporating lower-scale characteristics from the transmitter side with high-scale characteristics from the decoder side. In the UNet3P architecture, Decoder Stage 1 combines the characteristics map from Encoder Phase 1 (same scale), Decoder Phases 2, 3, and 4, and the bridge connection (large-scale). The characteristics map from Encoder Step 1, Encoder Stage 2, Decoder Stages 3, 4, and the bridge are combined in Decoder Stage 2 (large scale). The information from the first two stages of the encoder (at a lower scale), the third stage of the encoder (at the same scale), the fourth stage of the decoder, and the bridge are combined in the third stage of the decoder (large scale). Stage 4 of the decoder combines the information from stages 1–3 of the encoder (smaller scale), stage 4 of the encoder (same scale), and the bridge. The UNet3P architecture is depicted as a block diagram in Figure A2.
